# Simple‐Challenging‐Difficult (SCD) Difficulty Classification for Horizontal Bone Augmentation

**DOI:** 10.1111/jerd.70116

**Published:** 2026-01-26

**Authors:** Cheng‐Hsiang Hsu, Andrea Laureti, Zhaozhao Chen, Istvan A. Urban, Alessandro Pozzi, Hom‐Lay Wang

**Affiliations:** ^1^ Private Practice Taipei Taiwan; ^2^ Department of Periodontics and Oral Medicine University of Michigan School of Dentistry Ann Arbor Michigan USA; ^3^ Division of Fixed Prosthodontics and Biomaterials University of Geneva Geneva Switzerland; ^4^ Department of Chemical Science and Technologies University of Rome Tor Vergata Rome Italy; ^5^ Urban Regeneration Institute Budapest Hungary; ^6^ Department of Clinical Science and Translational Medicine University of Rome Tor Vergata Rome Italy; ^7^ Department of Restorative Sciences The Dental College of Georgia at Augusta University Augusta USA; ^8^ Department of Restorative Dentistry and Biomaterials Sciences Harvard School of Dental Medicine Boston Massachusetts USA

**Keywords:** bone defect morphology, dental implants, guided bone regeneration, horizontal bone augmentation

## Abstract

**Objective:**

To present a novel difficulty classification for horizontal bone augmentation (HBA) grounded in site‐specific morphometric characteristics, aiming to assist clinicians in achieving predictable guided bone regeneration (GBR).

**Overview:**

HBA is often needed after tooth loss for proper implant placement. GBR is reliable, but success and complexity depend on defect shape. The new Simple–Challenging–Difficult (SCD) classification sorts HBA by defect type and existing bone support: intraosseous defects are within native bone, while extraosseous require regeneration beyond it. Simple cases have horizontal and vertical support; Challenging ones lack horizontal but keep vertical support and need ≤ 4 mm extraosseous augmentation; Difficult defects lack both supports or require > 4 mm extraosseous augmentation.

**Conclusions:**

The SCD classification provides clinicians with a structured approach to assess defect morphology, enabling more accurate treatment planning, better anticipation of potential complications, and improved patient outcomes in horizontal bone regeneration procedures. Clinicians are encouraged to incorporate this classification into their routine assessment protocols to support informed decision‐making and to maximize treatment safety and efficacy.

**Clinical Significance:**

Precise evaluation of defect morphology and quantification of residual osseous support are essential for planning the optimal regenerative technique. The proposed classification system provides a systematic framework to facilitate a more consistent and predictable management of horizontal alveolar ridge augmentation.

## 1 Introduction

Adequate alveolar bone is essential for achieving complete circumferential osseointegration of dental implants [1, 2, 3]. After tooth loss, physiological bone remodeling results in both horizontal and vertical ridge resorption [4, 5]. Additional factors including periodontal or endodontic infections, sinus pneumatization, trauma, or congenital agenesis may also contribute to alveolar bone deficiencies [6]. Consequently, ridge augmentation procedures are commonly required to restore sufficient bone volume and facilitate optimal implant placement [7]. Horizontal bone augmentation (HBA) can be performed using a variety of surgical approaches, including autogenous block grafting, guided bone regeneration (GBR) with either titanium‐reinforced barriers or resorbable membranes, titanium meshes, and ridge expansion techniques [7, 8, 9, 10, 11]. The effectiveness of these techniques has been demonstrated by the high survival rates of implants placed in regenerated bone, consistently exceeding 95% [12].

Clinical studies on GBR for HBA have consistently demonstrated long‐term outcomes with implant success rates comparable to those obtained in native bone [12, 13, 14, 15, 16, 17, 18]. Evidence indicates that the extent of horizontal gains attained through GBR is influenced by both the surgical techniques [19] and biomaterial utilized [20]. Conventional GBR protocols employing particulate grafts with absorbable membranes generally yield mean horizontal augmentation gain between 1.5 and 3.8 [21, 22, 23]. In contrast, innovative methods applied to cases of severe ridge atrophy have reported horizontal gains ranging from 5.0 to 7.0 mm [10, 24, 25]. Although GBR techniques have demonstrated documented efficacy, the predictability of outcomes is primarily influenced by local anatomical factors and defect morphology [20]. More contained defects, bounded by the adjacent bone contour, enable the application of less complex and more reliable surgical techniques [26, 27, 28]. In accordance with the “PASS” principles for predictable bone regeneration, these defects support optimal angiogenesis, graft stability, and facilitate primary wound closure [27]. However, the management of horizontal alveolar ridge deficiencies may range from relatively straightforward to highly complex scenarios. While decision trees have been proposed to assist in selecting the most appropriate surgical technique [7, 29, 30] and in determining whether GBR should be performed simultaneously or prior to implant placement [8], to date, no structured system has been introduced to formally classify the difficulty of HBA procedures. The present model extends the Simple‐Challenging‐Difficult (SCD) framework, initially developed for vertical bone augmentation (VBA) [31] with the aim of providing clinicians with a systematic approach to guide treatment planning, anticipate potential challenges of HBA procedures, and facilitate clinical decision‐making to improve predictability. The present paper specifically focuses on GBR techniques for HBA, as membrane‐based GBR has been shown to achieve bone gain comparable to block grafting while offering reduced invasiveness and lower postoperative complication rates [32].

## 2 Proposed Difficulty Classification and Case Illustration

The proposed SCD classification categorizes HBA into three levels of surgical complexity, based primarily on the extent of residual horizontal and vertical bony support at the augmentation site. Within this framework, HBA defects, defined as morphological discrepancies between the existing alveolar ridge and the ideal prosthetically driven bone volume, are classified as intraosseous or extraosseous depending on whether regeneration remains confined within or extends beyond the native bone contour.

**FIGURE 1:**
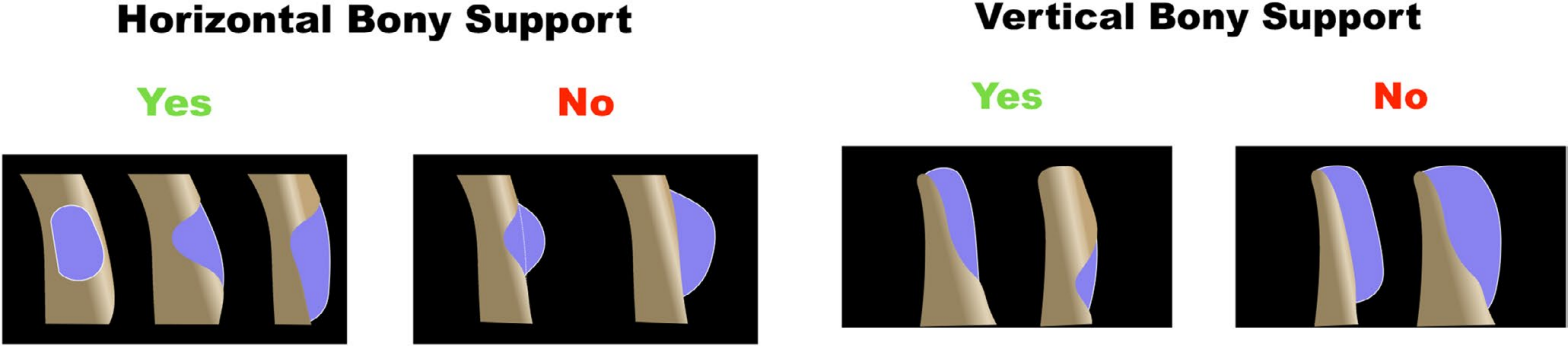
Schematic representation of horizontal bone augmentation (HBA) illustrating the presence or absence of residual horizontal and vertical bony support on the axial and cross‐section view, respectively. Intraosseous defects correspond to grafting performed entirely within the native bone envelope, where the graft remains contained within the original ridge profile. Extraosseous defects require grafting beyond the native bony contour and are non‐contained by definition. Vertical bony support refers to the apico‐coronal basal portion of the alveolar ridge that may serve as a stabilizing reference for graft placement and membrane fixation. When vertical support is absent, graft stability relies entirely on the barrier membrane and fixation devices. Within this framework, the configuration of residual horizontal and vertical bony support determines graft stabilization requirements and overall surgical difficulty, as defined by the present SCD classification.

Intraosseous defects involve graft containment within the residual native bone profile, thereby providing horizontal bony support. Extraosseous defects require regeneration beyond the native bone envelope and are therefore non‐contained, regardless of remaining vertical bone. Here, residual vertical bone may provide stabilization for the graft and membrane fixation, but not containment, lowering technical challenge when compared to defects lacking both horizontal and vertical support. The configuration of residual bony support strongly influences graft stability and overall technical difficulty (Figure 1). As horizontal support is lost or the extraosseous component increases, graft stabilization becomes progressively more demanding, reaching its highest complexity when neither horizontal nor vertical containment is present (Table 1).

**TABLE 1:** The Simple–Challenging–Difficult (SCD) classification for horizontal bone augmentation (HBA) is based on the configuration of residual bony support in the horizontal and vertical dimensions of the alveolar ridge, as well as on the extent of the horizontal extraosseous component.

	Horizontal support	Vertical support	Horizontal extraosseous component	Defect and graft stability	Surgical approach
Simple	Yes	Yes	0 mm	Well‐contained defect with complete bony walls ensuring stable graft housing	GBR with resorbable membrane; fixation might not be needed; simultaneous implant placement possible if primary stability achieved
Challenging	No	Yes	≤ 4 mm	Partially contained defect with limited buccal extension; vertical wall provides residual support for graft stabilization	GBR with fixated absorbable membranes or reinforced barriers; staged implant placement approach preferred
Difficult	No	No^a^	> 4 mm	Non‐contained defect with minimal or no residual bony support; graft stability depends entirely on membrane or mesh fixation	GBR requiring rigid space maintenance (titanium‐reinforced barriers or titanium mesh) or fixated collagen membranes; staged implant placement indicated

*Note*: Simple defects present both horizontal and vertical bony support and correspond to a fully intraosseous configuration within the native ridge contour. Challenging defects lack horizontal but retain vertical bony support, with a horizontal extraosseous component not exceeding 4 mm. Difficult defects are characterized by the absence of effective horizontal and vertical bony support or by a horizontal extraosseous component exceeding 4 mm.

^a^ In selected cases, limited vertical bony support may still be present; however, when the horizontal extraosseous extension exceeds 4 mm, the defect behaves morphologically as a fully non‐contained configuration and is therefore classified as Difficult, requiring a staged implant placement approach.

Within this conceptual framework, the SCD classification provides clinicians with a structured method to assess the morphological difficulty of horizontal GBR based on defect configuration.

From a clinical perspective, the SCD classification is intended to: (1) be applied during preoperative planning to support defect assessment, (2) guide treatment planning, (3) support clinical decision‐making.

Regarding implant placement timing in horizontal guided bone regeneration. During preoperative assessment, clinicians integrate clinical examination with three‐dimensional (3D) radiographic evaluation to classify the defect according to the SCD system. This morphological classification provides an estimate of the anticipated surgical difficulty by accounting for residual horizontal and vertical bony support and the extent of horizontal augmentation required. Based on the assigned SCD category, clinicians can plan the regenerative approach more effectively, including the selection of appropriate barrier membranes or space‐maintaining devices and the choice between simultaneous or staged implant placement.

The following subsections describe each category (Simple, Challenging, and Difficult) and illustrate representative clinical cases.

### 2.1 Simple Horizontal Defect (HS)

**FIGURE 2:**
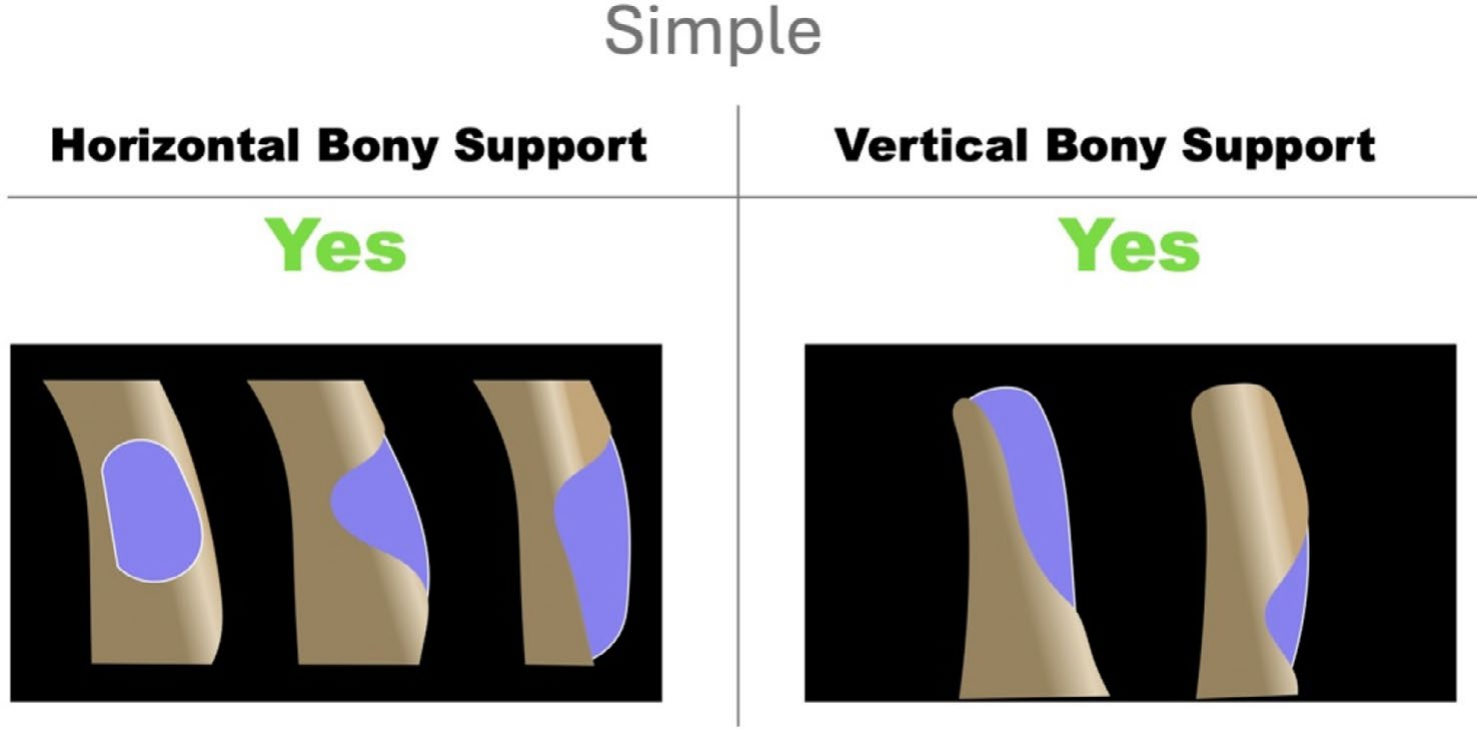
Simple horizontal defect (HS). Defects characterized by the preservation of both horizontal and vertical bony support, resulting in a fully intraosseous configuration. The graft remains contained within the native ridge profile, allowing for predictable stabilization of the regenerative space.

In HS cases, the graft is confined within the boundaries of the residual alveolar ridge, remaining inside the vestibular contour of the adjacent bone; consequently, the HBA is fully intraosseous. HS offers both horizontal and vertical support to the graft (Figure 2). As a contained defect, GBR can be easily stabilized since the augmented region is supported by the surrounding bony walls. In these situations, the use of a bioresorbable membrane in combination with a particulate bone substitute is generally considered the treatment of choice; fixation pins might be optional [8, 14]. Furthermore, if primary implant stability can be obtained in the correct prosthetic implant position [33], simultaneous implant placement during HBA may also be appropriate [8].

**FIGURE 3:**
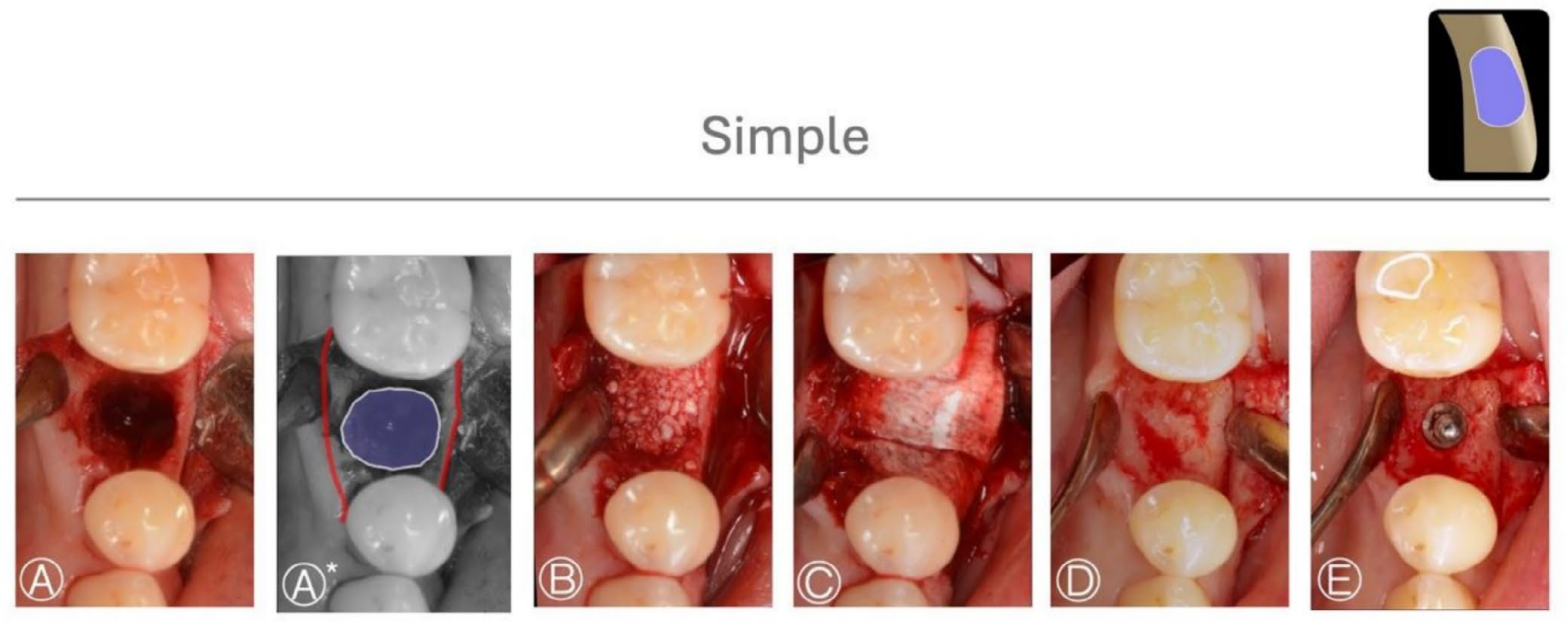
*Case 1*: Simple HBA case. (A, A*) Occlusal view of the bone defect, with a graphic overlay illustrating the area to be regenerated (violet) and the native bone contour (red). (B, C) Guided bone regeneration performed using a particulate bone graft (allograft) covered with a collagen membrane. (D) Occlusal view of the ridge after 6 months of healing. (E) Occlusal view of the implant placed in the regenerated bone.

**FIGURE 4:**
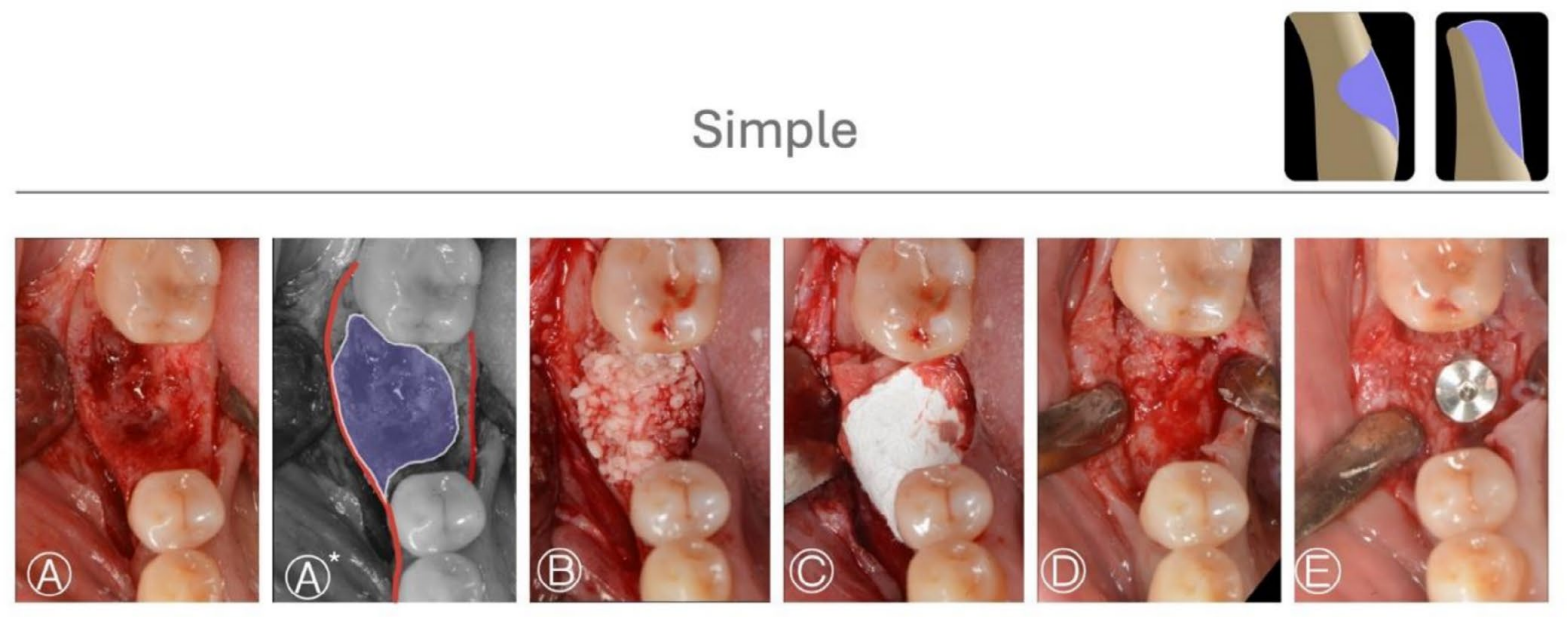
*Case 2*: Simple HBA case. (A, A*) Occlusal view of the bone defect with a graphic representation of the planned horizontal bone augmentation (HBA); the native bone contour is indicated in red, and the area to be regenerated is shown in violet. (B, C) Guided bone regeneration performed using a particulate bone graft (allograft) covered with a collagen membrane. (D) Occlusal view of the ridge after 4 months of healing. (E) Occlusal view of the implant placed in the regenerated bone.

**FIGURE 5:**
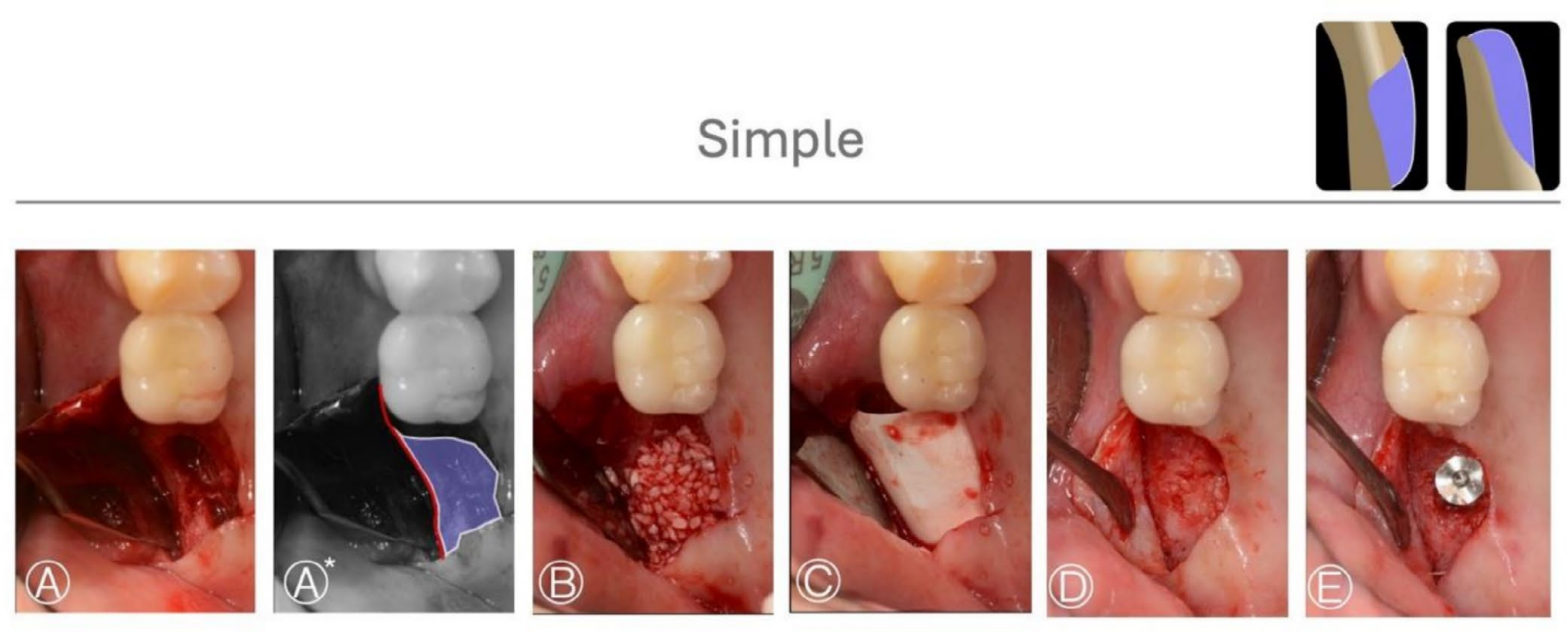
*Case 3*: Simple HBA case. (A, A*) Occlusal view of the bone defect. (B, C) Guided bone regeneration performed using a particulate bone graft (allograft) covered with a collagen membrane. (D) Occlusal view of the ridge after 6 months of healing. (E) Occlusal view of the implant placed in the regenerated bone.

Case 1 (Figure 3) shows a simple HBA case treated with GBR by bone graft (allograft) and a collagen membrane in a fresh extraction socket defect. A periapical X‐ray taken 5 months after the procedure showed adequate condition for implant placement. Case 2 and 3 (Figures 4 and 5) demonstrated HS buccal defects. These cases were treated with GBR, using a bone graft (allograft) and a collagen membrane. The re‐entry procedure after healing for implant placement revealed significant bone gain.

### 2.2 Challenging Horizontal Defect (HC)

**FIGURE 6:**
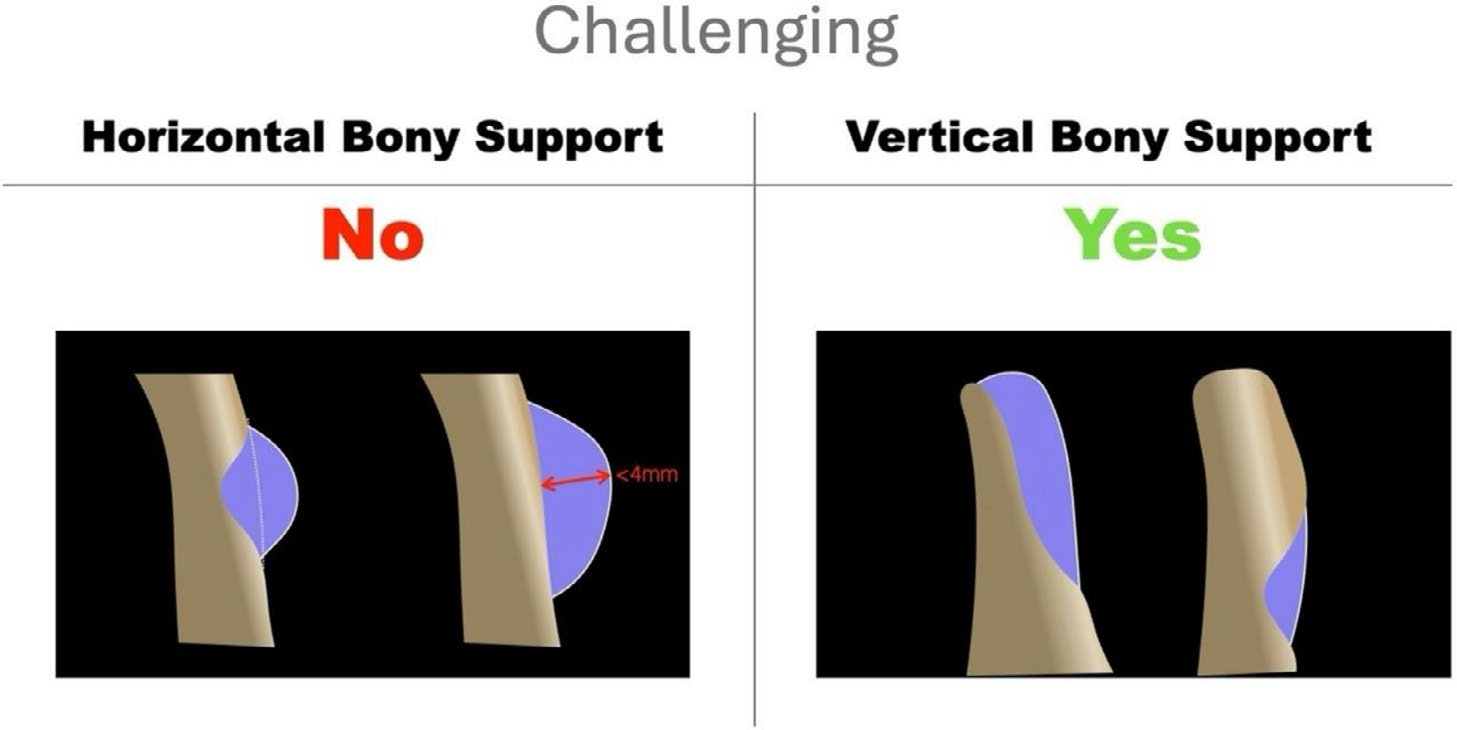
*Challenging*: Challenging horizontal defect (HC). Defects characterized by the preservation of vertical bony support and the absence of horizontal bony support, resulting in a partial or total extraosseous configuration. The horizontal extraosseous component does not exceed 4 mm, allowing graft stabilization with the bone in the apical part of the defect acting as a stabilizing reference.

When horizontal bony support is absent but vertical bony support is preserved, the defect is classified as HC. In these cases, regeneration extends beyond the native bone envelope and is therefore extraosseous and non‐contained by definition. However, the presence of residual vertical bony support provides a basal reference for graft placement and membrane fixation, contributing to improved graft stability. The horizontal extraosseous component should not exceed 4 mm (Figure 6). This value refers specifically to the horizontal extraosseous component and is intended as a pragmatic, morphology‐based clinical reference to stratify surgical difficulty, rather than as a strict biological cutoff.

**FIGURE 7:**
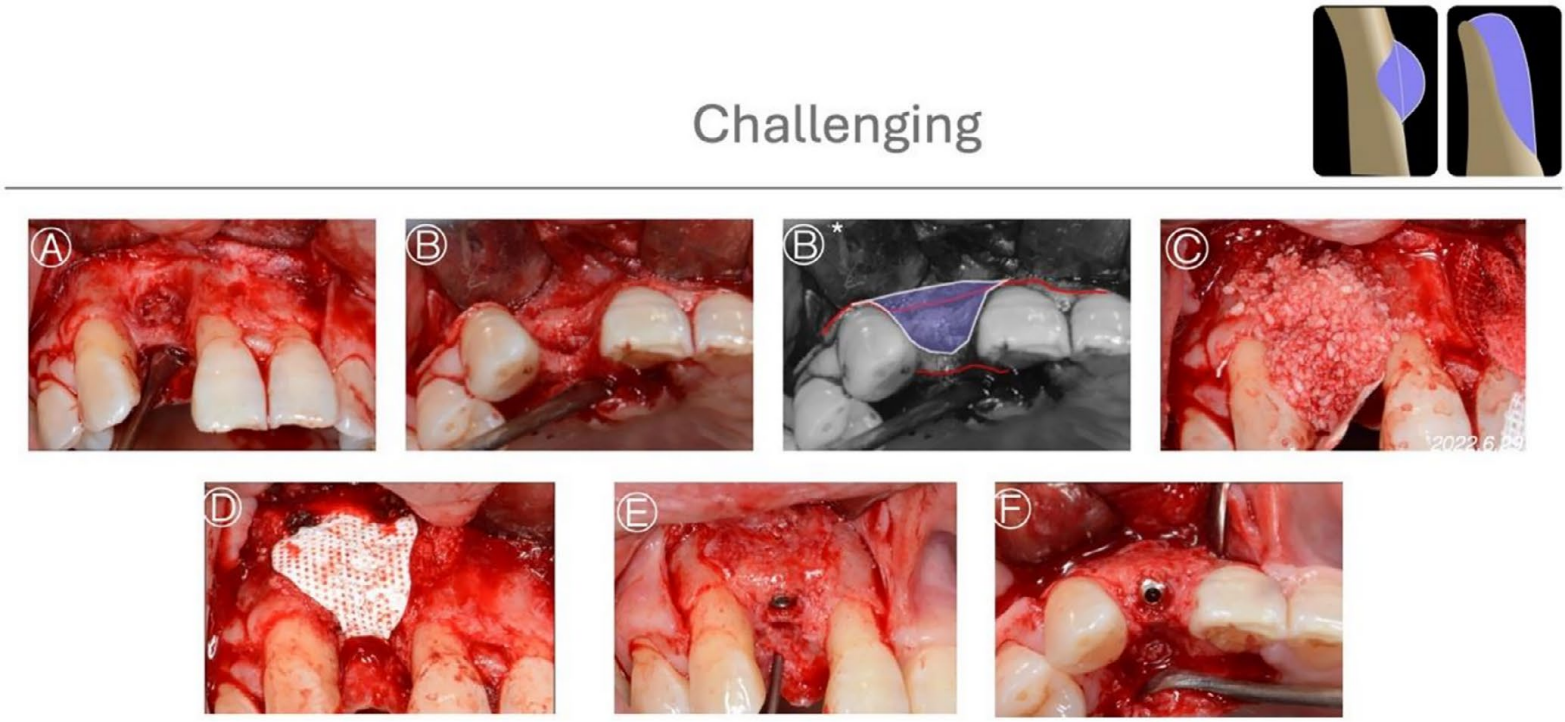
*Case 4*: Challenging HBA case. (A, B, B*) Buccal and occlusal views of the bone defect, characterized by the absence of horizontal bony support with preservation of vertical support; the planned horizontal bone augmentation is highlighted by graphic annotation. (C, D) Guided bone regeneration performed using a particulate bone graft (allograft) covered with a non‐resorbable titanium‐reinforced membrane. (E, F) Buccal and occlusal views of the ridge after 8 months of healing and staged implant placement.

**FIGURE 8:**
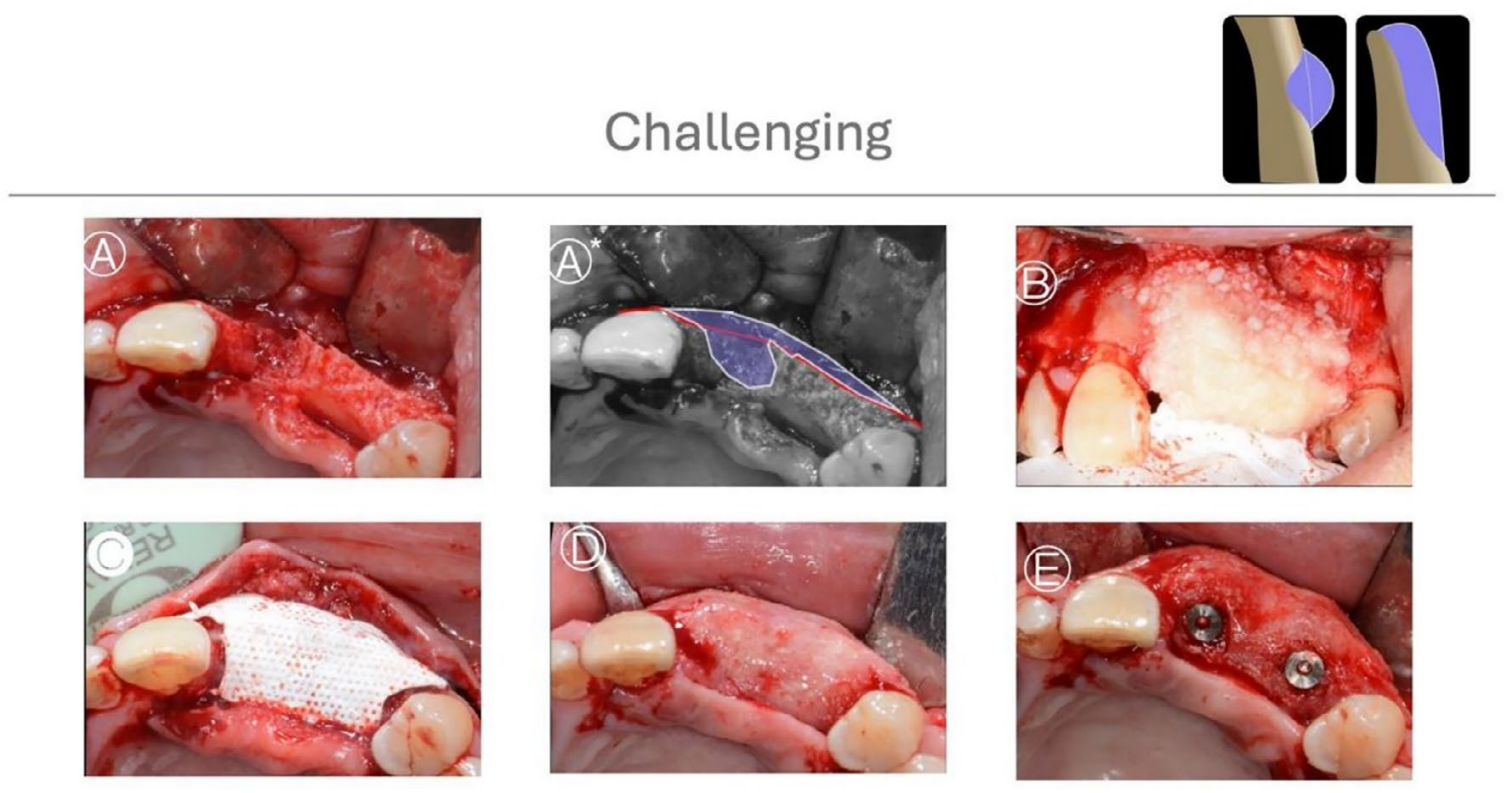
*Case 5*: Challenging HBA case. (A, A*) Preoperative buccal view showing a horizontal ridge deficiency in the anterior maxilla, with a graphic overlay illustrating the planned horizontal bone augmentation. (B, C) Buccal view of guided bone regeneration performed using a particulate bone graft (allograft) covered with a titanium‐reinforced membrane. (D, E) Occlusal views of the ridge after 6 months of uneventful healing and implant placement.

Under these conditions, GBR can be performed using resorbable membranes or titanium‐reinforced non‐resorbable barriers stabilized with fixation pins. A staged approach is generally recommended, although simultaneous implant placement may be considered in selected cases with adequate primary stability and advanced surgical expertise [8] (Figures 7 and 8).

### 2.3 Difficult Horizontal Defect (HD)

**FIGURE 9:**
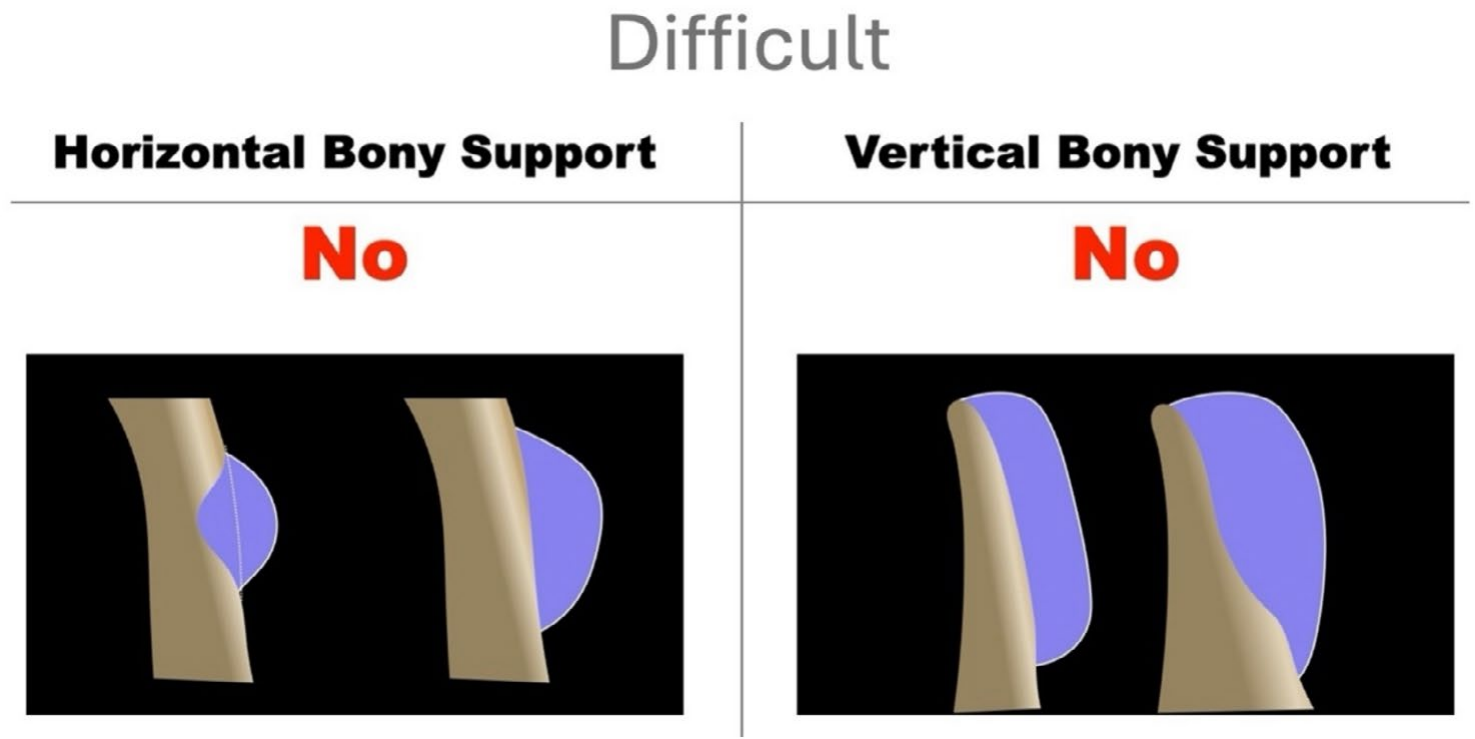
Difficult horizontal defect (HD). Defects characterized by the absence of both horizontal and vertical bony support, resulting in a fully extraosseous configuration that requires graft stabilization entirely outside the native bone envelope.

HD defects are characterized by the absence of both horizontal and vertical bony support (Figure 9). In addition, defects presenting a horizontal extraosseous extension greater than 4 mm are classified as HD, even when limited vertical bone height is preserved. In such cases, the residual vertical bone does not provide effective stabilization, and the defect behaves morphologically as a fully non‐contained space.

**FIGURE 10:**
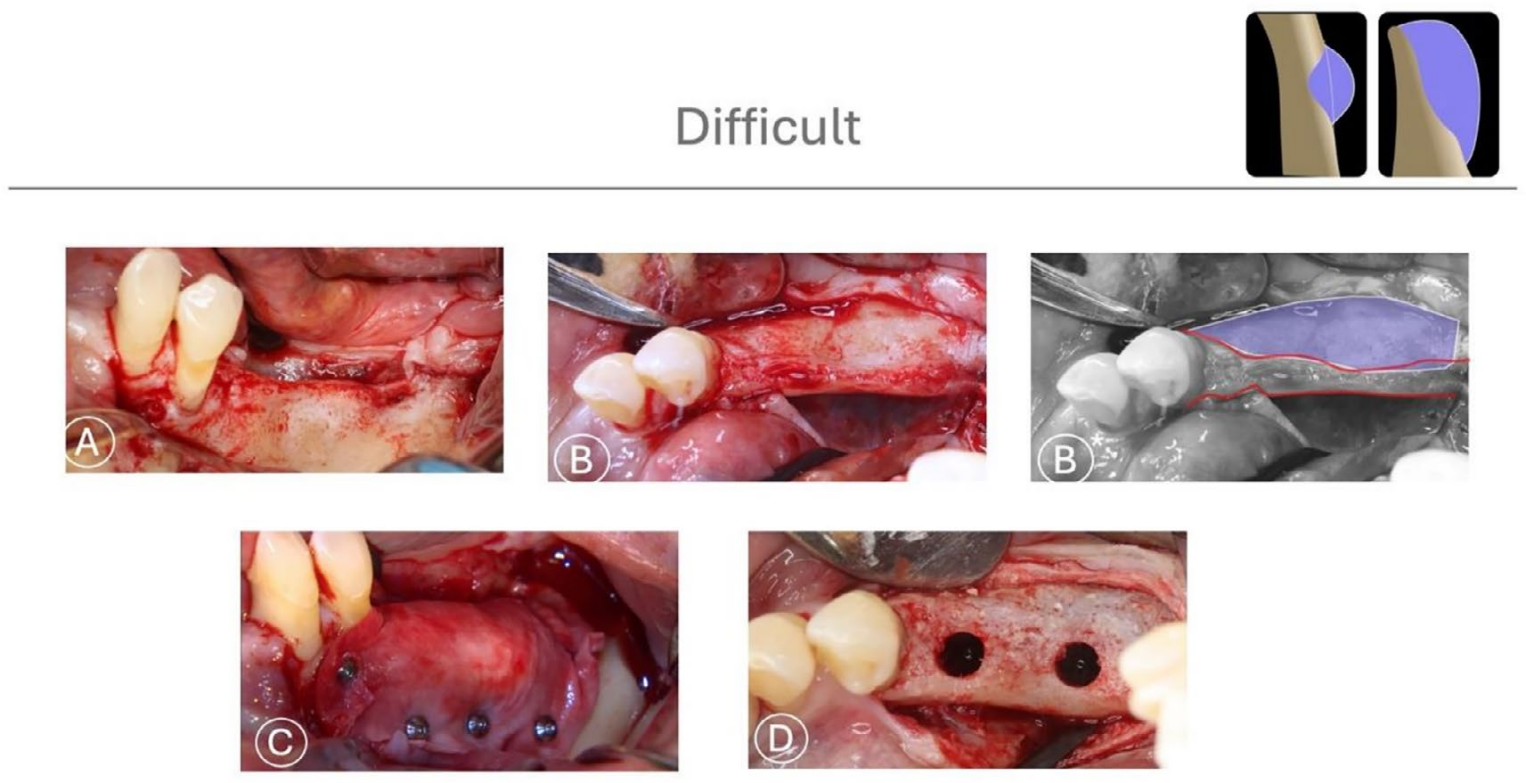
*Case 6*: Difficult HBA case. (A, B, B*) Buccal and occlusal views of a horizontal ridge deficiency in the mandible after full‐thickness flap elevation, with graphic annotation illustrating the planned augmentation. (C) Extraosseous bone graft covered with a collagen membrane stabilized with fixation pins. (D) After 6 months of uneventful healing and membrane removal, significant horizontal bone gain was observed, allowing for proper implant placement.

**FIGURE 11:**
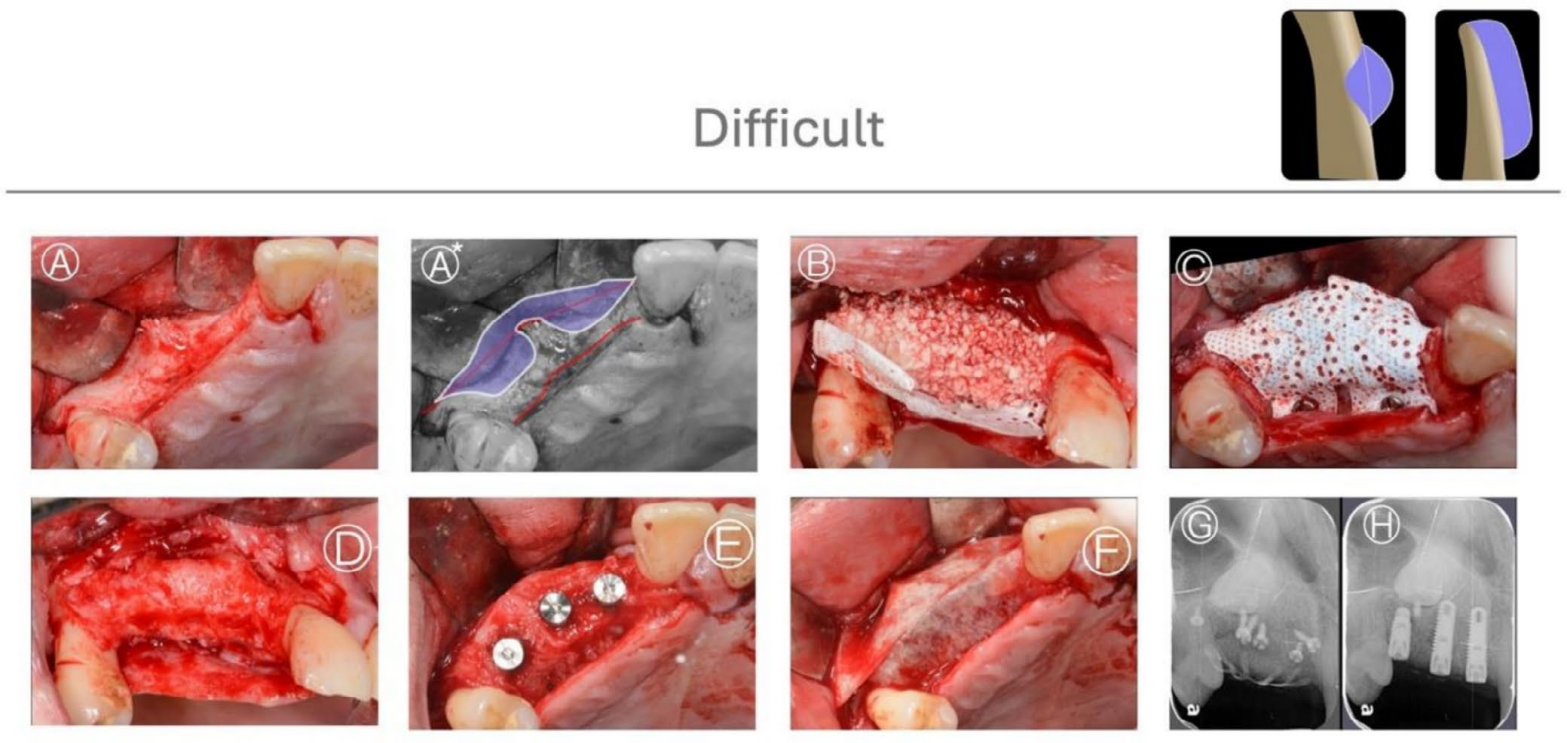
*Case 7*: Difficult HBA case. (A, A*) Buccal view of a horizontal ridge deficiency in the maxilla after full‐thickness flap elevation, with graphic annotation illustrating the defect configuration. (B) Guided bone regeneration performed using a particulate bone graft (allograft) covered with a perforated titanium‐reinforced membrane. (C) Occlusal view of the stabilized membrane. (D) After 6 months of uneventful healing and membrane removal, significant horizontal bone gain was observed. (E) Following implant placement, an additional GBR procedure using a resorbable membrane was performed to optimize ridge contour. (F) Postoperative periapical radiograph after GBR. (G, H) Postoperative periapical radiographs after implant placement.

In these cases, GBR must be performed entirely outside the existing bony contour, with graft stability relying exclusively on membrane fixation and adaptation. Such defects are among the most complex clinical challenges, as the lack of native bone support combined with the pressure exerted by the overlying soft tissues can significantly compromise graft stability. Under these circumstances, three viable options can be considered: absorbable membranes secured with fixation pins, non‐resorbable barriers stabilized with fixation pins, or titanium meshes and implant placement should only be considered following complete bone healing (Figures 10 and 11) [34, 35].

## 3 Discussion

Although classifications of horizontal defects and decision trees for treatment selection have been proposed in the literature [7, 8, 29], the surgical difficulty associated with these procedures has never been formally assessed. The SCD classification [31] introduces a structured framework to support clinicians in planning horizontal GBR while anticipating the challenges of HBA surgical procedures. The SCD classification should be interpreted as a morphology‐based planning and risk‐stratification framework within horizontal GBR. It is not intended to prescribe specific surgical techniques but rather to support clinical judgment by structuring defect assessment, guiding treatment planning, and informing decisions regarding graft stabilization and implant timing based on the expected complexity of the defect.

Horizontal ridge augmentation using guided bone regeneration (GBR) encompasses varying levels of complexity, from straightforward surgical procedures to highly intricate cases. Defect morphology serves as a crucial factor in this context; specifically, concave intraosseous defects offer an advantageous anatomical configuration that aids in stabilizing particulate graft material and minimizes the risk of displacement. Tsai et al. [26] underscored this concept through a clinical study assessing the stability of horizontal bone augmentation around implants.

Their findings demonstrated that bone contour augmentation produced more reliable outcomes in larger intraosseous defects (concavity greater than 2 mm), regardless of the grafting material employed.

The present classification is conceptually consistent with the system proposed by Tinti and Parma‐Benfenati [28] for peri‐implant defects, which categorizes bone defects based on whether they are contained within or extend beyond the envelope of adjacent bone. The authors emphasized that the presence of surrounding bony walls and the resultant morphological stability are critical factors influencing both the predictability of treatment outcomes and the complexity of surgical procedures.

The horizontal threshold of 4 mm proposed in the present classification was not derived from a single numerical cutoff reported in the literature and should not be interpreted as a biological limit. Rather, it represents a pragmatic, morphology‐based reference introduced by the authors to facilitate standardization of surgical difficulty in horizontal GBR. Existing decision‐making frameworks for lateral ridge augmentation primarily consider the overall width of the residual ridge or the total amount of horizontal augmentation required, without explicitly accounting for the morphological distribution of the defect relative to the native bone envelope [7]. Clinically, defects of similar horizontal extent may behave very differently depending on how much of the augmentation remains intraosseous versus extraosseous.

Although this classification primarily addresses the defect morphology, biomaterial selection also plays a pivotal role in treatment planning. According to a network meta‐analysis by Calciolari et al. [20], resorbable collagen membranes in conjunction with bone grafts are recommended for small, space‐making defects exhibiting concavities greater than 2 mm (> 2 mm) within the bony contour. Conversely, larger non–space‐making defects with concavities < 2 mm or cases involving GBR outside the bony envelope, remain challenging and require stabilized barrier membranes to ensure graft stability and prevent displacement. Similarly, Buser et al. [36] concluded that collagen membranes are advantageous for managing small‐ to medium‐sized defects due to their ease of handling and low incidence of complication, whereas non‐resorbable, titanium reinforced membranes are more suitable in complex cases with extensive deficiencies. However, the latest developments in collagen membranes and surgical techniques have made their use feasible even in difficult cases [10].

As described in the text, simultaneous implant placement with HBA may also be feasible, even though technically more demanding. In the decision tree proposed by Yu et al. on simultaneous or staged lateral ridge augmentation, implant placement performed at the same time as HBA is predictable in smaller intraosseous defects (1–3 mm), whereas staged implant placement should be preferred in extraosseous defects or when more than 6 mm of width gain is required [7]. However, it must be highlighted that although certain defects may be classified as “simple” to regenerate, the addition of simultaneous implant placement significantly increases procedural complexity. Consequently, appropriate case selection in accordance with the clinician's expertise is critical to ensure predictable outcomes.

Beyond defect characteristics and biomaterial selection, systemic and local factors strongly influence GBR outcomes. Conditions such as hyperglycemia and smoking may impair wound healing and increase the risk of postoperative infection [36]. Meticulous soft tissue management is equally critical: ensuring sufficient keratinized mucosa, appropriate flap thickness, and careful release from muscular attachments are essential steps to achieve tension‐free primary closure, which is crucial for predictable healing particularly in challenging (HC) and difficult (HD) HBA cases [34] Finally, although the effectiveness of GBR in HBA is well documented, limitations remain. A meta‐analysis by Naenni et al. on the efficacy of lateral bone augmentation before implant placement [18] reported that in 6 of 25 studies, after healing, either additional augmentation was required at implant placement or narrower implants were used than initially planned, underscoring that horizontal augmentation techniques may not always fully restore sufficient bone volume. Therefore, clinicians should anticipate possible limits of augmentation procedures and plan accordingly by, for example, preparing for potential secondary grafting procedures, considering the use of alternative implant sizes, or employing staged treatment protocols to address insufficient bone volume.

Limitations of the proposed SCD classification should be acknowledged. First, the SCD classification is based on morphological considerations and clinical experience; it has not yet been prospectively validated against clinical outcomes or complication rates.

Second, it does not quantify the total volume of bone regeneration required, nor does it aim to predict the absolute amount of bone gain that can be achieved for individual cases. Third, additional factors influencing clinical complexity—such as patient‐specific anatomical variations, soft tissue quality, local infection, or systemic health conditions—are not directly incorporated into the classification.

Several classification systems and decision‐making frameworks for alveolar ridge augmentation have been proposed in the literature, primarily focusing on defect dimensions, ridge width, or the selection and timing of surgical techniques. While these approaches provide valuable guidance for treatment planning, they generally do not specifically address the morphological difficulty of horizontal bone augmentation procedures, nor do they differentiate between intraosseous and extraosseous defect configurations. The proposed SCD classification does not aim to replace existing classification schemes or decision trees. Rather, it complements them by introducing a morphology‐based stratification of surgical difficulty, grounded in the extent of residual horizontal and vertical bony support. By focusing on graft containment and stabilization rather than on defect size alone, the SCD framework addresses a gap in the current literature and provides an additional layer of information to support preoperative planning and clinical communication in horizontal GBR.

## 4 Conclusion

The Simple‐Challenging‐Difficult classification offers clinicians a practical tool to plan horizontal bone regeneration, anticipate surgical challenges, and improve predictability according to defect morphology. By tailoring treatment strategies to defect morphology, clinicians can enhance patient comfort, reduce complications, and achieve more predictable long‐term results. Clinical decision‐making should integrate these parameters with professional expertise to ensure the safest and most effective treatment outcomes.

## Author Contributions

**Cheng‐Hsiang Hsu:** involved in conception, design; preparing the cases; analysis and interpretation of cases; initial and final drafting of the work; final approval of the version to be published, accountable for all aspects of the work. **Andrea Laureti, Zhaozhao Chen, Alessandro Pozzi, Istvan A. Urban,** and **Hom‐Lay Wang:** involved in conception, design; analysis and interpretation of case; initial and final drafting of the work; final approval of the version to be published, accountable for all aspects of the work, and accountable for all aspects.

## Funding

The authors have nothing to report.

## Conflicts of Interest

Drs. Urban and Wang occasionally serve as speakers for Osteogenics Biomedical and have received honoraria. Additionally, Dr. Wang has been awarded a research grant through the University to conduct a clinical trial.

## Data Availability

The data that support the findings of this study are available from the corresponding author upon reasonable request.
